# Nanoparticulate apatite and greenalite in oldest, well-preserved hydrothermal vent precipitates

**DOI:** 10.1126/sciadv.adj4789

**Published:** 2024-01-26

**Authors:** Birger Rasmussen, Janet R. Muhling, Nicholas J. Tosca

**Affiliations:** ^1^ School of Earth Sciences, University of Western Australia, 35 Stirling Highway, Perth, WA 6009, Australia.; ^2^ Department of Earth Sciences, University of Cambridge, Cambridge CB2 3EQ, UK.

## Abstract

Paleoarchean jaspilites are used to track ancient ocean chemistry and photoautotrophy because they contain hematite interpreted to have formed following biological oxidation of vent-derived Fe(II) and seawater P-scavenging. However, recent studies have triggered debate about ancient seawater Fe and P deposition. Here, we report greenalite and fluorapatite (FAP) nanoparticles in the oldest, well-preserved jaspilites from the ~3.5-billion-year Dresser Formation, Pilbara Craton, Australia. We argue that both phases are vent plume particles, whereas coexisting hematite is linked to secondary oxidation. Geochemical modeling predicts that hydrothermal alteration of seafloor basalts by anoxic, sulfate-free seawater releases Fe(II) and P that simultaneously precipitate as greenalite and FAP upon venting. The formation, transport, and preservation of FAP nanoparticles indicate that seawater P concentrations were ≥1 to 2 orders of magnitude higher than in modern deepwater. We speculate that Archean seafloor vents were nanoparticle “factories” that, on prebiotic Earth, produced countless Fe(II)- and P-rich templates available for catalysis and biosynthesis.

## Teaser

Oldest seafloor vent precipitates point to hydrothermal growth of Fe(II) and P nanoparticles in nutrient-rich early oceans.

## INTRODUCTION

High-temperature hydrothermal alteration of basaltic crust is a global process that emits hot, acidic, metal-rich fluids into today’s oxygenated ocean. The vent-derived Fe(II) commonly precipitates from plumes as polymetallic sulfide particles or as oxyhydroxides following rapid oxidation in seawater. The dissolved (<200 nm) and particulate (>450 nm) Fe fractions may be carried for >4000 km in distal hydrothermal plumes (*1*). The scavenging of dissolved P by settling Fe-oxyhydroxide particles is an important sink of seawater P (*2*, *3*), which can preserve a high-fidelity sedimentary record of changes in oceanic P (*4*).

In the early Precambrian, hydrothermal venting is thought to have been the main source of Fe in banded iron formations (BIFs) and jaspilites (*5*, *6*). Most models for BIF and jaspilite deposition invoke upwelling of dissolved Fe(II)-rich deeper water followed by biological oxidation in the surface ocean (*7*, *8*), either indirectly involving molecular oxygen produced by Cyanobacteria or directly by microaerophilic Fe-oxidizing bacteria or anoxygenic phototrophs. As with modern hydrothermal sediments, the oxidation of Fe(II) and its settling through the water column is considered to be a major pathway for the deposition of seawater P. These models rely on the widely held assumption that the primary Fe phases were Fe(III)-oxyhydroxides; however, this is now being debated following recent studies of BIFs that suggest that the earliest phases were Fe(II)-silicates (*9*–*13*).

**Fig. 1.**
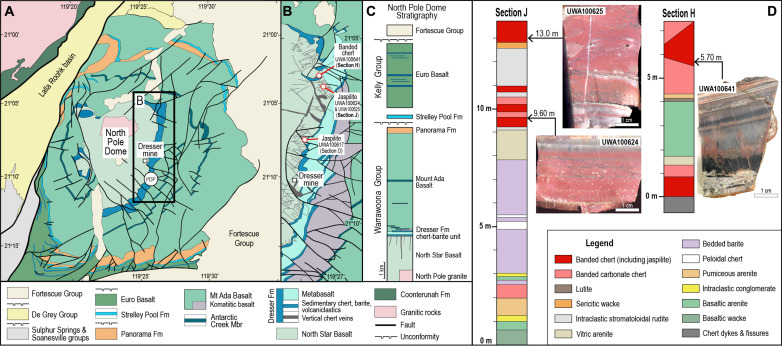
Locality map and stratigraphic column, North Pole Dome. (**A**) Simplified geological map of the North Pole Dome area. PDP, Pilbara Drilling Project. (**B**) Map of the eastern flank of the dome showing the location of stratigraphic sections and samples. (**C**) Simplified stratigraphic column. Fm, formation. (**D**) Stratigraphic columns for sections H and J of Buick (*20*) showing location of jaspilitic cherts (UWA100624 and UWA100625) and thinly laminated chert (UWA100641).

Unlike BIFs, jaspilites are common in the oldest well-preserved successions and are ideal for investigating the mineralogy of the primary plume particles because they are siliceous, vent-proximal deposits with a relatively simple mineral paragenesis. Jaspilites, which are Si-rich and Fe-poor (<15 wt % Fe) chemical sediments, contain minute particles of hematite that is largely thought to have formed by biological oxidation of vent-derived Fe(II)(aq), thereby providing evidence for the early rise of oxygenic photosynthesis (*14*) or anoxygenic photosynthesis (*15*). The oldest well-preserved jaspilites occur in the ~3.5-billion-year (Ga) Dresser Formation, a basalt-dominated succession from the North Pole Dome area, Pilbara Craton, Australia (Fig. 1). Recent studies of the Dresser jaspilites found that the sole primary iron precipitate was hematite that formed from microbially deposited ferric oxides/hydroxides (*15*, *16*), although doubts were raised (*17*). The absence of Fe(II)-rich phases such as greenalite was attributed to its purported solubility in acidic to near-neutral seawater (*15*, *16*).

To investigate the hydrothermal precipitates in vent-proximal exhalites, we carried out in situ high–spatial resolution transmission electron microscopy (TEM) study of exceptionally well-preserved red (jaspilite), white, and gray banded chert from the ~3.5-Ga Dresser Formation (Fig. 1). Our TEM observations reveal that submicron-sized particles of greenalite, siderite, and fluorapatite (FAP) co-occur with less abundant hematite. The greater abundance of hematite particles in jaspilites that have undergone more intense secondary oxidation challenges the interpretation that the oxide is a primary precipitate. Geochemical modeling of hydrothermal alteration of seafloor basalts by anoxic, sulfate-free seawater predicts that greenalite and FAP are among the main precipitates during hydrothermal fluid–seawater mixing. Their formation under similar temperature ranges and stability windows potentially explains their ubiquitous co-occurrence in plume-derived deposits. The non-dissolution of FAP nanoparticles in ferruginous, acidic to near-neutral seawater implies that the shallow ocean was enriched in dissolved P by one to two orders of magnitude compared to today, with implications for the paradigm of the P-limited Archean biosphere. Looking further back in time, our results from the oldest well-preserved exhalative deposits offer insights into one of the prime sites for life’s emergence: hydrothermal vents. Basalt-hosted seafloor vents may have acted as nanoparticle “factories” producing plumes of countless templates for the assembly of different RNA sequences (*18*), a precondition for RNA-based evolution (*19*).

### Samples and geological setting

Samples of chert and jaspilite were collected from the chert-barite unit of the Dresser Formation, Warrawoona Group (Fig. 1), which in the North Pole area contains mainly pillow basalts and minor ultramafic volcanic rocks. The mafic-dominated volcanic pile is intercalated with thin banded chert beds (typically <10-m thick), although the chert-barite unit is locally up to 50-m thick and extends laterally for >20 km (*20*–*22*). The chert-barite unit is enclosed in metamorphosed basalts composed of pillowed and massive flows as well as subvolcanic sills. The contacts between the chert-barite unit and upper and lower basalts are concordant and non-erosional, indicating that deposition and extrusion were not marked by major hiatuses. On the basis of the abundance and size of vesicles in a pillow basalt immediately below the chert-barite unit, water depths of ≤100 m have been inferred (*23*). More recently, it has been proposed that some of the banded cherts are geyserites that formed in terrestrial hot springs and ponds (*24*).

The chert-barite unit comprises silicified mafic epiclastic mudstones and sandstones, beds of barite, and banded chert interpreted to include primary hydrothermal precipitates (*23*). In this study, we examined samples of red, white, and gray banded chert from the Dresser Formation along the eastern flank of the North Pole Dome, where it comprises thin sequences of shallowly dipping (<45°) volcano-sedimentary rocks (Fig. 1). The mineral assemblages in the underlying basalts indicate prehnite-pumpellyite to greenschist facies metamorphism (*25*). Ar-Ar radiometric dating of illite around chert veins in the underlying basalt has been interpreted to indicate hydrothermal fluid flow at ~3.25 Ga, ~3.06 Ga, and ~2.29 Ga (*26*), whereas Sm-Nd isochrons from jaspilites in the chert-barite unit suggest postdepositional modifications at ~2.26 Ga (*27*).

The banded cherts, which vary in color from red to white and gray, tend to be only a few meters thick (<5 m) and are intercalated with silicified volcaniclastic sedimentary rocks and bedded barite (Fig. 1D). It is generally agreed that the banded iron–bearing cherts are exhalative deposits from seafloor hydrothermal vents (*15*, *20*). The underlying basalts are transected by numerous black chert veins that terminate in the chert-barite unit. The black chert veins and associated black chert beds contain carbonaceous structures that have been compared to fossilized microbes (*28*–*30*), although their biological affinity is unclear (*31*–*33*).

Although the primary precipitate of red cherts (jaspilites) is generally thought to be ferrihydrite or hematite (*15*, *16*), the primary chemical sediment in gray and white banded chert is less clear. To investigate the primary sediments of the ferruginous banded cherts, we prepared polished thin sections of samples of jaspilite (UWA100617, UWA100624, and UWA100625) and bedded gray chert (UWA100641) from the chert-barite unit of the ~3.5-Ga Dresser Formation (Fig. 1). For comparison, we also examined polished thin sections of bedded chert, including jaspilite, from the 3.46-Ga Marble Bar Chert Member, intersected in Archean Biosphere Drilling Project drill-hole #1 (ABDP1) (*34*), and Neoarchean ferruginous chert and BIF from drill-hole ABDP9 in the Hamersley Province in Western Australia (*9*) and drill-holes GKP01 and GKF01 in Griqualand West, Kaapvaal Craton, South Africa (*10*).

## RESULTS

### Nanoscale characterization

#### Red banded chert (jaspilite; UWA100624 and UWA100625)

**Fig. 2.**
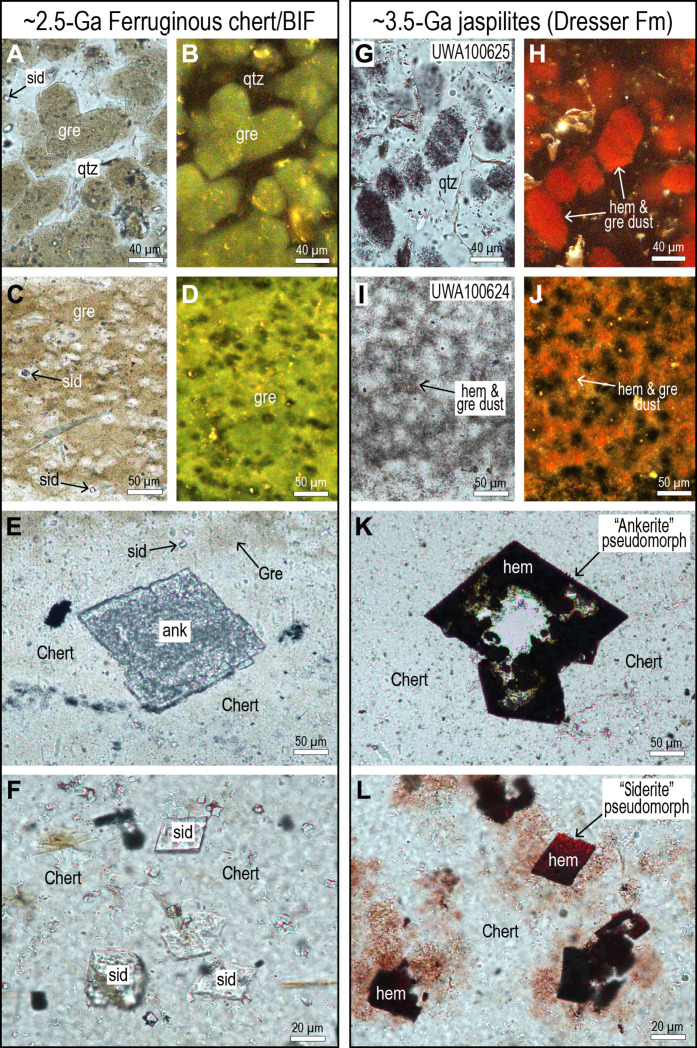
Optical light images comparing jaspilite from Dresser Formation with ~2.5-Ga ferruginous chert and BIF. (**A**) Plane-polarized light (PPL) image of polygonal structures defined by minute particles of greenalite (gre) and siderite (sid) in fine-grained quartz (qtz). (**B**) Incident light (IL) image of minute greenalite (gre) particles surrounded by quartz (qtz). (**C**) PPL image of “splotchy” chert composed of dusty greenalite (gre) and small siderite rhombs (sid). (**D**) IL image showing the presence of greenalite dust (light green) in chert. (**E**) PPL image of large ankerite rhomb (ank) in laminated chert with greenalite dust (gre) and tiny siderite crystals (sid). (**F**) PPL image of numerous small siderite rhombs (sid) in chert. (**G**) PPL image of polygonal structures defined by minute opaque hematite particles in chert. (**H**) IL image of minute hematite (red) particles surrounded by quartz. (**I**) PPL image of splotchy chert defined by minute opaque hematite (hem) particles. (**J**) IL image showing the presence of hematite particles (red). (**K**) PPL image of large rhomb-shaped crystal (formerly ankerite) replaced by hematite (hem). (**L**) PPL image of numerous small rhomb-shaped crystals (formerly siderite) replaced by hematite (hem). [(A) and (B)] Drill-hole GKP01, drill depth of 222.2 m (Kuruman Iron Formation); [(C) to (F)] drill-hole GKF01, drill depth of 369.0 m (upper Nauga Formation); [(G) to (L)] Dresser Formation.

Light microscopy of jaspilitic cherts from section J (Figs. 1 and 2, A to D) shows that they comprise mainly fine-grained quartz (5 to 20 μm) interspersed with minute particles of hematite (<2 μm). The jaspilites also contain pseudomorphs of larger, rhomb-shaped crystals (up to 200 μm) as well as smaller rhombs (10 to 50 μm) that have been replaced by iron oxides and quartz (Fig. 2, E and F). On the basis of their morphology and habit and comparison with non-oxidized drill-core samples of ferruginous chert (Fig. 2, K and L), the rhombs are interpreted to have originally been ankerite-dolomite and siderite, respectively.

The fine-grained hematite locally defines polygonal structures separated by inclusion-free chert (Fig. 2, A and B), as well as mottled textures (Fig. 2, C and D) interpreted to be silica dehydration or shrinkage structures (*35*). Similar structures are common in jaspilites from the 3.46-Ga Marble Bar Chert (*34*, *36*) and in younger ferruginous cherts (2.60 Ga to 2.45 Ga) from the Transvaal and Hamersley regions, where the polygons are mainly defined by greenalite nanoparticles (Fig. 2, G and H) (*9*, *10*, *36*). In plane-polarized light, the hematite dust appears to be the only phase in the chert; however, using reflected light at high magnification (×500), abundant light gray particles are visible between the more conspicuous hematite particles.

**Fig. 3.**
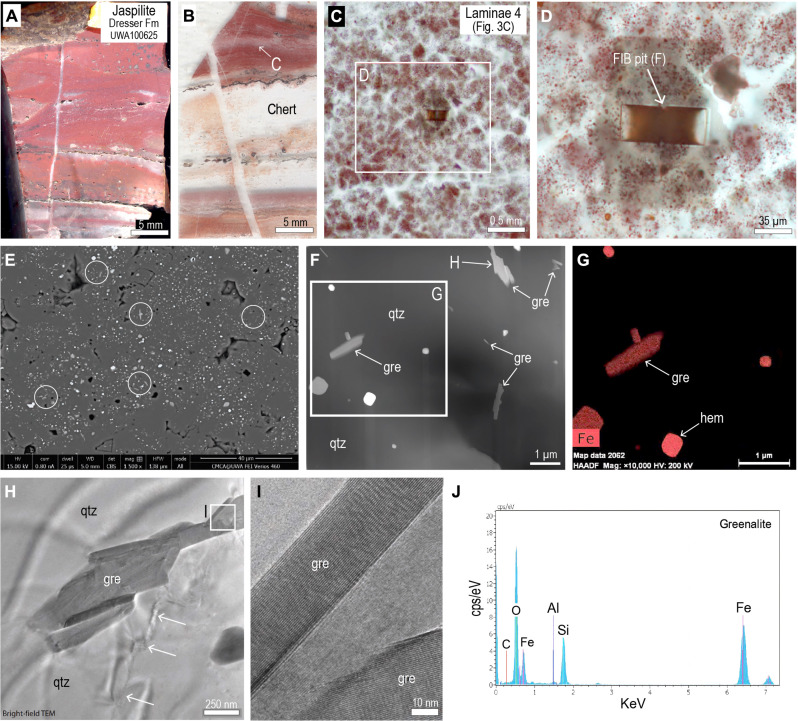
Optical and electron microscope images of jaspilite from Dresser Formation (UWA100625). (**A**) Hand specimen of jaspilite from section J. (**B**) Polished thin section of jaspilite showing hematite-rich chert and hematite-poor chert. (**C**) PPL image of jaspilitic chert with polygonal pattern. (**D**) PPL image showing location of focused ion beam (FIB) pit from jaspilitic chert. (**E**) Backscattered electron image of jaspilitic chert showing abundant minute particles, including platy laths (circled). HV, high voltage. (**F**) High-angle annular dark-field (HAADF) scanning TEM (STEM) image of FIB foil (D) showing abundant greenalite laths (gre) and equant hematite (white) in chert cement. (**G**) STEM-EDS iron element map. Mag, magnification. (**H**) Bright-field TEM image of greenalite (gre) nanoparticles in chert from foil cut by FIB. (**I**) High-resolution TEM image of greenalite particle with characteristic structural modulations. (**J**) TEM–EDS spectra from greenalite nanoparticle in FIB foil of greenalite-rich chert.

**Fig. 4.**
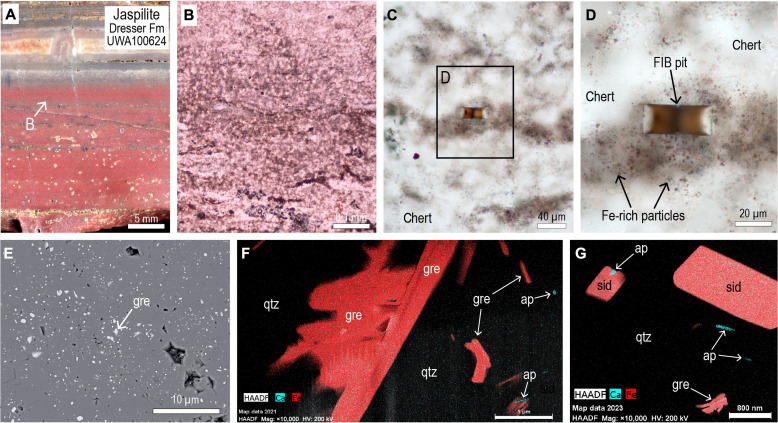
Optical and electron microscope images of jaspilite from Dresser Formation (UWA100624). (**A**) Hand specimen of jaspilite from section J. (**B**) PPL image of jaspilite showing splotchy chert defined by heterogeneous distribution of minute Fe-rich particles. (**C** and **D**) PPL images showing location of FIB pit from jaspilitic chert. (**E**) Backscattered electron image of jaspilitic chert showing abundant minute particles, including platy laths (gre). (**F** and **G**) STEM-EDS iron and calcium element maps from FIB foil of jaspilitic chert showing minute crystals of greenalite (gre), siderite (sid), and apatite (ap) in chert cement (qtz).

Examination by scanning electron microscopy–energy-dispersive x-ray spectroscopy (SEM-EDS) shows the presence of abundant light gray platy and equant particles interspersed between hematite grains (Figs. 3 and 4). TEM of foils (8 μm by 8.5 μm) removed by focused ion beam (FIB) milling from the jaspilitic chert (Figs. 3, A to D, and 4, A and D) shows that hematite is less abundant than other Fe-rich particles (Figs. 3, F to H, and 4, F and G). Many of the platy particles (up to 1-μm long and 0.1-μm wide) are randomly oriented and comprise mainly Fe, Si, and O, with minor Al (Fig. 3J). The platy crystals have a (001) *d*-spacing of ~0.73 nm and a 2.2-nm modulated superlattice characteristic of greenalite (Fig. 3I). The greenalite occurs as single small plates as well as larger aggregates (Figs. 3H and 4F) comprising multiple plates that have a combined length of up to 3 μm. Also in the chert are equant particles (0.5 to 2.0 μm in size) comprising mainly Fe, C, and O, consistent with siderite (Fig. 4G). Hematite, which occurs as subhedral to rounded particles 0.1 to 2.0 μm in size, is the least abundant iron phase in the FIB foils (Figs. 3 and 4).

Scattered among the larger greenalite and siderite particles are tiny prismatic crystals (50- to 400-nm long) (Fig. 4, F and G), composed of Ca, P, and O with a minor F, which are interpreted to be FAP. The FAP prisms are randomly oriented and uniformly distributed in the chert, although some also abut greenalite and siderite crystals (Fig. 4, F and G). TEM-EDS element maps of FIB foils show that the P is strictly colocated with Ca, indicating that FAP is the main mineralogical host of the P in the jaspilites.

#### Gray banded chert (UWA100641)

**Fig. 5.**
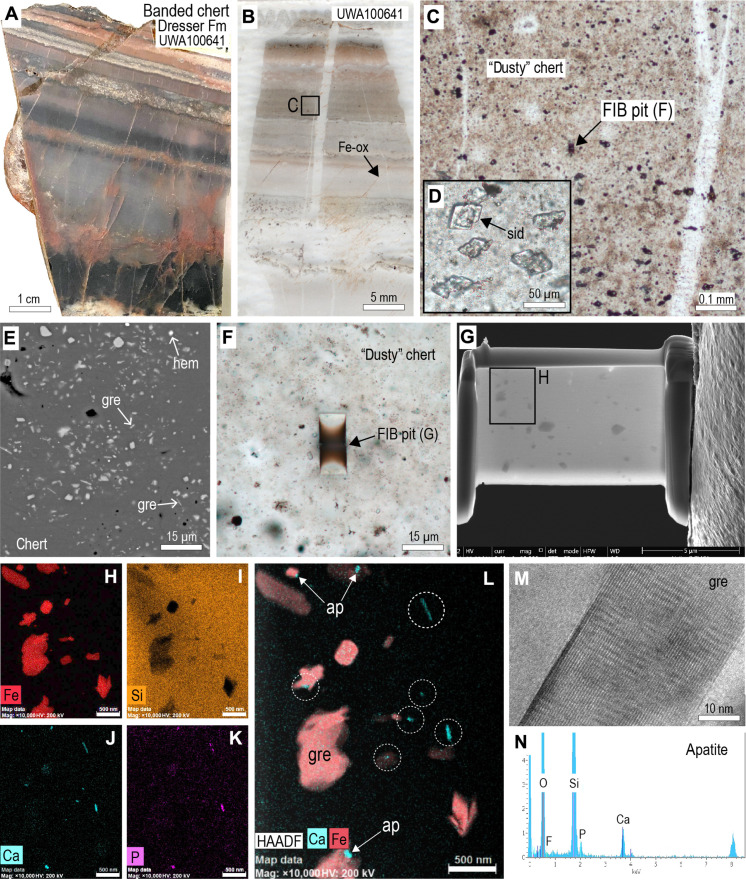
Optical and electron microscope images of laminated gray chert from Dresser Formation (UWA100641). (**A**) Hand specimen of gray banded chert from section H. (**B**) Polished thin section of thinly laminated chert showing hematite-poor laminae. (**C**) PPL image showing location of FIB pit from “dusty” chert. (**D**) PPL image of small siderite (sid) rhombs in dusty chert. (**E**) Backscattered electron image of dusty chert showing abundant minute particles of greenalite (gre) and siderite, and rare hematite (hem). (**F**) PPL image showing location of FIB pit in dusty chert. (**G**) Secondary electron image of FIB foil removed from pit in (F). (**H** to **L**) STEM-EDS images for iron (H), silicon (I), calcium (J), phosphorus (K), and combined iron-calcium (L) element maps. (**M**) High-resolution TEM image of greenalite particle with characteristic structural modulations. (**N**) TEM-EDS from apatite nanoparticle in FIB foil of greenalite-rich chert.

Samples of gray banded chert from section H (Figs. 1 and 5) also preserve thin laminae (<1 mm) that contain abundant micron-sized platy and blocky particles that are near-transparent in plane-polarized light and light gray in reflected light. Backscattered electron (BSE) imaging shows lath- and rhomb-shaped grains consistent with greenalite and siderite while hematite particles are rare, comprising <1% of the dust-sized particles (Fig. 5E). Unlike the jaspilites, the gray chert bands preserve abundant pyrite euhedra (5 to 20 μm) and siderite rhombs (20 to 50 μm in length) (Fig. 5D). The partial preservation of macroscopic siderite and pyrite indicates that these chert bands have undergone less secondary oxidation than the jaspilites from section J (UWA100624 and UWA100625).

TEM and analysis of FIB foils removed from the gray chert (Fig. 5, C, F, and G) confirm that the light gray particles identified in reflected light and BSE images are greenalite and siderite. The greenalite, which occurs as randomly oriented plates (≤0.75-μm long and ≤0.1-μm wide), comprises mainly Fe, Si, and O, with minor Al (Fig. 5, H to L). It has well-developed structural modulations along crystal edges, typical of greenalite (Fig. 5M). The more equant particles (0.5 to 1 μm in size) comprise Fe, C, and O, with minor Ca, consistent with siderite. Hematite, which is the least abundant iron phase in the banded chert, was not intersected in the FIB foils from this sample.

Among the larger greenalite and siderite particles are abundant minute particles (10- to 300-nm long) composed of Ca, P, and O, with minor F (Fig. 5, J to L and N), which are interpreted to be FAP. As in the jaspilites, the FAP grains are randomly oriented and evenly distributed in the chert and locally abut greenalite and siderite grains (Fig. 5L). The minute bright specks in TEM-EDS P element maps correspond with elevated Ca (Fig. 5, J and K), indicating that the P in the banded cherts is hosted by FAP nanoparticles.

### Phase equilibrium constraints on the behavior of P in basalt-hosted hydrothermal systems

To examine the behavior of dissolved phosphorus in subseafloor hydrothermal systems during the Archean, we estimated equilibrium solution compositions and thermodynamically predicted mineralogy by executing reaction path models. These models, originally developed by Tosca and Tutolo (*13*), were modified to include P species and minerals, as described below. The models are designed to represent three major components of subseafloor-hosted hydrothermal systems: (i) the reaction zone, where peak pressure and temperature conditions are encountered near the base of the near-axis hydrothermal system; (ii) the upflow and reaction of hydrothermal fluids through, and with, the oceanic crust; and (iii) the mixing between hydrothermal vent fluids and ambient seawater.

Extensive observational, experimental, and theoretical constraints on reaction zone conditions shows that water/rock ratios are generally low (on the order of 1), which allows reaction between basalt/gabbro and primary and secondary minerals to buffer several aspects of fluid chemistry. At the pressure/temperature conditions relevant to Archean near-axis systems, which are expected to have featured shallower magma chamber depths and lower pressures of water-rock interaction, experimental and theoretical studies indicate that fluid chemistry is dominantly controlled by fluid-mineral equilibria involving feldspar, chlorite, epidote, quartz, and NaCl-rich fluids at approximately 375° to 400°C and 300 to 500 bar, which is consistent with geophysical constraints on pressure and temperature. We examined this system under rock-buffered conditions at 400°C and at 400 and 500 bar, which are taken to be broadly representative of Archean subseafloor reaction zone conditions on the basis of considerations discussed above (*13*).

Under these reaction zone conditions, previous work has shown that even at micromolar concentrations of sulfate (*6*), its effective absence allows the redox state of the Fe-O-S subsystem to be controlled by fayalite-magnetite-pyrrhotite-quartz equilibria. In addition, under these conditions, the abundance of plagioclase expected in Archean mid-ocean ridge–type basalts (*37*), combined with relatively low water/rock ratio, strongly buffers in situ pH to values close to 5 (*38*). As Tosca and Tutolo (*13*) have noted, major deviations from this in situ pH would require large changes to plagioclase composition in Archean mid-ocean ridge–type basalts, which is not supported by current petrologic and geochemical considerations. A significant role for calcic plagioclase is also supported by strong positive Eu anomalies that have been recovered from a wide variety of Archean sedimentary rocks (*39*). Last, given the constraints on reaction zone conditions described above, we specify a modern seawater Cl concentration (0.55 mol/kg), which allows us to examine completely buffered fluid chemistry in the Na_2_O-K_2_O-CaO-MgO-FeO-Fe_2_O_3_-Al_2_O_3_-SiO_2_-P_2_O_5_-H_2_O-HCl-H_2_S-HF system as a function of Ca concentration.

To examine the behavior of phosphorus under reaction zone conditions, we added phosphorus as a component in the calculations, specifying equilibrium with respect to either hydroxyapatite or FAP in the presence of a P-bearing aqueous fluid. Although experimental and observational data on the behavior of P under reaction zone conditions is relatively limited, this assumption is supported by reports of accessory and vein apatite in lower sheeted dykes inferred to have formed under conditions ranging from 400 to 700 bar and >425°C (*40*, *41*). The inclusion of FAP also requires constraints on dissolved F concentrations. Although the behavior of dissolved F in near-axis subseafloor hydrothermal systems is not well understood, observational data have shown that F is strongly depleted in mid-ocean ridge hot spring fluids relative to modern seawater (*42*). Fluid-mineral equilibria suggest that aqueous F concentrations might become enriched under reaction zone conditions, but observational data clearly show that this is not the case (*42*, *43*). Rather, Seyfried and Ding (*43*) suggested that minor incorporation of F in multiple alteration phases such as epidote, actinolite, talc, and/or apatite may represent a collective set of sinks that serve to deplete aqueous F under such conditions. Thus, as a conservative estimate and in the absence of appropriate constraints on F concentration of Archean seawater, several of our calculations use a total dissolved F concentration of 70 μmol/kg, which is approximately equal to the modern marine value. In equilibrium with FAP, concentrations lower than this translate into higher total phosphate concentrations.

The second component of the reaction path models investigated phosphorus behavior in systems approximating those likely to be encountered as reaction zone fluids ascend through sheeted dyke complexes and higher permeability lavas that characterize the oceanic crust. We repeated calculations performed by Tosca and Tutolo (*13*), which involved separation of the hydrothermal fluid from the rock buffer and fluid upflow through mafic rocks along a prescribed cooling and decompression pathway from 400°C/400 bar to 250°C/250 bar, approximately consistent with conditions associated with venting of hydrothermal fluids at the modern seafloor.

This system also included the effects of reaction between the hydrothermal fluid and hypothetical wall rocks within the oceanic crust (*13*, *44*), which involved reaction with varying masses of fresh crystalline basalt chosen to represent average modern mid-oceanic ridge–type basalt (MORB). This allowed us to evaluate the effect of water/rock ratio (from 1 to 5000), which, for modern subseafloor systems and ancient hydrothermally altered equivalents, is inferred to have ranged between approximately 250 to 1000 based on minor, trace element, and isotope geochemistry (*45*–*49*).

Although observational data on the behavior of P during the hydrothermal alteration of dykes and lavas associated with hydrothermal upflow are limited, we designed the reaction path models to incorporate constraints on the average abundance of P present in modern MORB. Available data indicate that the average P_2_O_5_ content of MORB is ~0.18 wt %, which is largely hosted by olivine where P_2_O_5_ content can range from 0.2 to 0.4 wt % (*50*). We incorporated this constraint into our model of hydrothermal upflow conditions by adding phosphorus to the olivine that was reacted with hydrothermal fluids at varying water/rock ratio. For our nominal model runs, we chose an olivine content of 1 mol % P (equivalent to 0.4 wt % P_2_O_5_) (*50*). Upon reaction between mid-ocean ridge basalt and cooling hydrothermal fluids, this P was released to the fluid where precipitation as FAP and/or hydroxyapatite may be possible, subject to thermodynamic saturation state.

The final component of the model evaluated mixing between hydrothermal fluids with ambient seawater. This involved cooling and decompression along H_2_O vapor saturation (Psat) from 250°C/250 bar, which is broadly representative of the conditions of venting for modern near-axis systems. Ambient seawater chemistry was designed in accordance with Tosca and Tutolo (*13*), where Na, K, Mg, and Cl were set to modern values and [SO_4_] was set to 0; [SiO_2_(aq)] was set to saturation with respect to amorphous silica. Seawater was anoxic and [Fe_Total_] was set to 100 μmol/kg, which is a conservative estimate on the basis of experimentally determined mineral solubility relationships (*51*). Values for [Ca], dissolved inorganic carbon, and pH were chosen to remain consistent with Ca-isotope constraints on carbonate chemistry (*52*) where [Ca] = 10 to 20 mmol/kg, [DIC] (Dissolved Inorganic Carbon) = 5 to 15 mmol/kg, and pH = 6.5 to 7.5.

We also examined the effect of marine [PO_4Total_] on the mineral assemblage produced upon venting, where precipitation of FAP or hydroxyapatite may be favored. Although low-temperature precipitation of phosphate minerals from seawater has been observed to involve metastable precursor phases such as octacalcium phosphate (OCP) largely due to inhibition by seawater Mg, it is likely that these kinetic effects may have been minimized under the elevated temperatures associated with mixing between hydrothermal vent fluids and ambient seawater. Specifying phosphate mineral precipitation by FAP as opposed to OCP or carbonate-FAP serves as a conservative estimate for phosphate concentrations because this is the least soluble of the apatite-mineral group end-members.

We used identical sources of thermodynamic data to those by Tosca and Tutolo (*13*) and implemented the reaction path models in Geochemists Workbench v2022. Thermodynamic data were generated with the Python module pyGeochemCalc (pyGCC), using the same data sources and algorithms as described by Tosca and Tutolo (*13*). The apparent standard molal Gibbs free energies of aqueous solutes, including dissolved phosphate species, were calculated according to the revised Helgeson-Kirkham-Flowers (HKF) equation of state (*53*) with modifications to aqueous Fe-chloro complexes described by Tosca and Tutolo (*13*). The standard molal Gibbs free energy of FAP and hydroxyapatite were calculated using data published by Zhu and Sverjensky (*54*) (fig. S1).

### Reaction zone conditions

Pressure serves as an important first-order control on fluid composition in equilibrium with altered basalt/gabbro at reaction zone conditions (*6*, *13*). Specifically, at in situ pH close to 5.0, the effective absence of seawater sulfate leads to reducing fluids characterized by significant total dissolved Fe content, whereas increases in pressure from 400 to 500 bar lead to significant decreases in Fe concentration (fig. S2).

Equilibrium between the aqueous fluid and either FAP (at modern seawater F concentrations) or hydroxyapatite results in very low equilibrium total phosphate concentrations and is largely a function of decreasing solubility of apatite-group minerals with increasing temperature. Total phosphate concentrations in the fluid range between 0.03 and 0.0001 μmol/kg (fig. S2). Although there are few experimental constraints on phosphate concentrations of subseafloor reaction zones, these results suggest that such systems may serve as a sink for P, rather than a source.

### Upflow of hydrothermal fluids through the oceanic crust

Reaction path models examining the cooling and decompression of reaction zone fluids (fig. S3) and their subsequent reaction with basaltic rocks present within the oceanic crust indicate that appreciable phosphate may be derived from these fluid-rock interactions (fig. S4). Tosca and Tutolo (*13*) showed that cooling and decompression of reaction zone fluids results in significant pH decreases that arise largely from destabilization of major aqueous metal chloro-complexes at decreasing temperature and pressure. Our models show that decreasing pH, in addition to decreasing temperature, together increase the solubility of apatite-group minerals, largely preventing their precipitation under some conditions. We find, however, that the phosphate concentration of hydrothermal upflow fluids is largely controlled by fluid/rock ratio, in addition to the total P content of basalt. At high fluid/rock ratios, phosphate release into the fluid is solely a function of the total P content of the basalt; under a large range of ratios (i.e., from approximately 250 and higher), P concentration increases linearly with decreasing water/rock ratio. This continues until low fluid/rock ratio conditions result in sufficient buffering of acidity by silicate hydrolysis to result in late-stage apatite saturation toward the lower temperature and pressure range of the reaction path (i.e., lower than approximately 300°C/300 bar). Under these conditions, total phosphate concentrations are also a function of fluoride concentration of the fluid, with decreasing values resulting in increasing total phosphate concentrations dictated through equilibrium between the fluid and FAP (fig. S5). In total, for fluid/rock ratios within the range of altered ophiolites and ancient hydrothermally altered rocks in upflow zones (i.e., 250 to 1000), the total phosphate concentration of the fluid ranges between 3 and 20 μmol/kg. This range is dependent on assumed fluoride concentrations, total P content of the mafic rocks interacting with the fluids, and by fluid/rock ratio.

### Hydrothermal vent fluid–seawater mixing

**Fig. 6.**
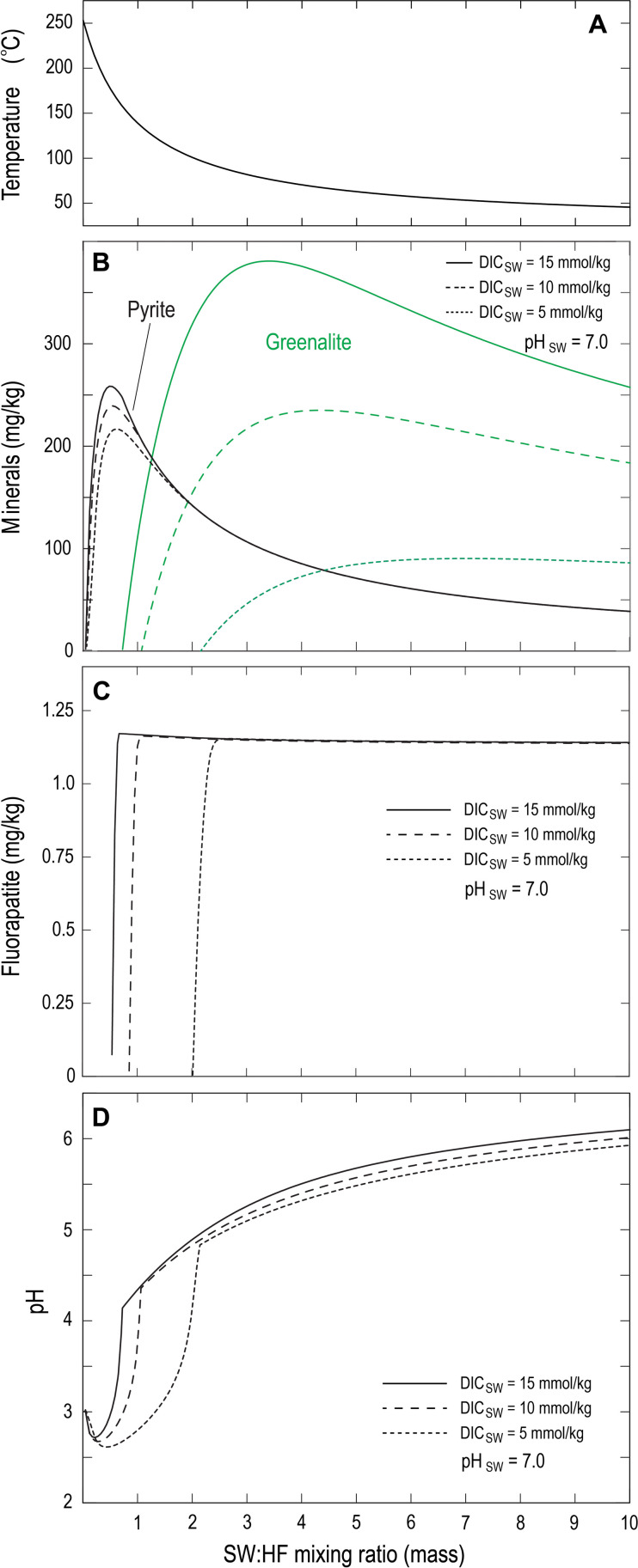
Reaction path calculations simulating the venting and mixing of high Fe/H_2_S fluids. Calculated temperature (**A**), mass of minerals precipitated (**B**), mass of FAP precipitated (**C**), and fluid pH (**D**) derived from the mixing between high Fe/H_2_S fluids (at 250°C and 250 bar; corresponding to those shown in figs. S3A and S4A) and anoxic SO_4_–free seawater at pH 7 and variable DIC concentrations. Minerals suppressed from the assemblage include quartz, chalcedony, coesite, cristobalite, dolomite, pyrrhotite, siderite, and minnesotaite. SW:HF, Seawater:Hydrothermal Fluid.

The results of reaction path models investigating the mixing between Archean seawater and hydrothermal fluids that have ascended through the oceanic crust indicate that FAP precipitation is thermodynamically predicted across a wide range of conditions. Reaction path models specifying relatively low dissolved phosphate concentrations of ~7.5 μmol/kg, corresponding to final concentrations derived from a fluid/rock ratio of 500 (fig. S5), show that FAP is predicted to precipitate at low mixing ratios between hydrothermal fluids and ambient seawater that correspond to a temperature range of ~150° to 175°C (Fig. 6). The early precipitation of FAP results mainly through an abrupt increase in fluid pH as acidic vent fluids and circum-neutral seawater mix; this leads to sharp increases in the concentration of deprotonated phosphate species, which increases FAP saturation driving its precipitation (Fig. 6). These results show that FAP and greenalite precipitate together across a narrow temperature range, representing overlapping stability windows, perhaps resulting from similarly nonlinear dependencies of saturation state on pH (*13*).

Tosca and Tutolo (*13*) also noted that the relative precipitation kinetics between greenalite and/or siderite may modulate the relative abundances of these two minerals precipitated upon mixing. This was examined by excluding siderite from a series of reaction path calculations, which simulates the effect of kinetic inhibitions on siderite precipitation on the final assemblage. We found that the abundance of FAP precipitated upon mixing shows no dependence on whether siderite is included in the mineral assemblage nor does it depend on whether the seawater has been conductively heated before mixing with hydrothermal fluids, as discussed by Tosca and Tutolo (*13*).

We also examined the effect of varying the total phosphate concentration of the hydrothermal fluid or ambient seawater on the timing and relative proportion of FAP precipitated upon mixing. Increases in the total phosphate concentration of the hydrothermal fluid result in negligible changes in the total proportion of FAP within the final assemblage. This is because, at total fluoride concentrations corresponding to modern (i.e., 70 μmol/kg), FAP precipitation occurs under a P-limited regime. The total amount of P contained within the hydrothermal fluid, however, is relatively low and is controlled only by a finite amount of P supplied by the vent fluid itself. Because of mixing with larger quantities of seawater, this results in relatively minor amounts of FAP that do not change upon mixing. We note, however, that the total concentration of aqueous phosphate within the hydrothermal fluid will necessarily be limited by any late stage FAP precipitation that might occur as hydrothermal fluids ascend through the oceanic crust (discussed above). In contrast to this behavior, increases in the total phosphate concentration of seawater result in corresponding increases in the proportion of FAP relative to other minerals in the assemblage such as greenalite (fig. S6).

### Paleoarchean seawater P concentrations

To constrain the minimum seawater P concentrations required to prevent FAP nanoparticle dissolution, we examined its solubility in seawater. Laboratory measurements of crystalline FAP solubility in modern seawater (with no Fe) at temperatures of 25° and 10°C, both imply that, even at pH 7, total P concentrations are on the order of 1 μmol/kg (fig. S7) (*55*, *56*). However, all estimates for the pH of seawater 3.5 Ga ago lie between about 6.5 and 6.75 (*52*, *57*, *58*). At these slightly lower pH values, total P would be even higher, by perhaps close to an order of magnitude.

**Fig. 7.**
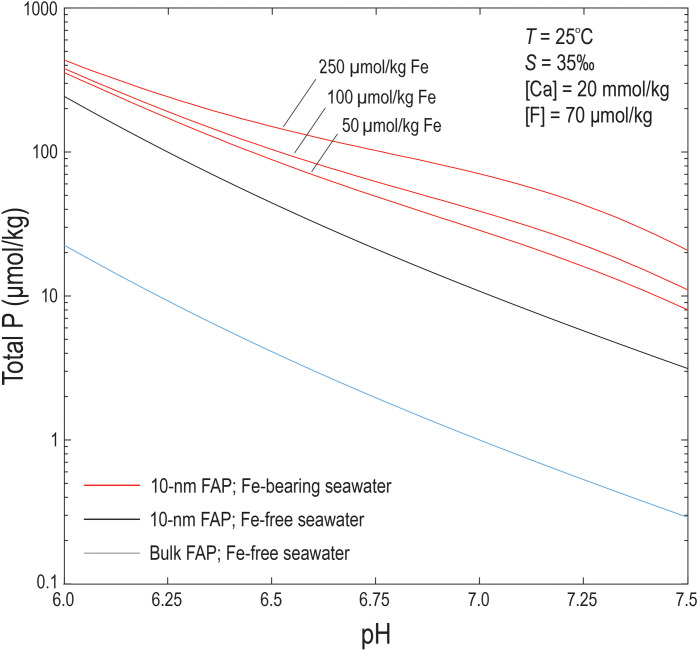
Solubility of FAP in seawater as a function of pH for a range of dissolved Fe^2+^ concentrations. Blue line corresponds to bulk FAP solubility in Fe-free seawater. Black line corresponds to the solubility of 10-nm spherical FAP particles in Fe-free seawater. Red lines correspond to the solubility of 10-nm spherical FAP particles in seawater with total dissolved Fe(II) concentrations of 50, 100, and 250 μmol/kg. Temperature (*T*) = 25°C, salinity (*S*) = 35 per mil (‰), total dissolved calcium of 20 mmol/kg, and total dissolved F of 70 μmol/kg.

However, given our constraints on the size of FAP nanoparticles deposited from Paleoarchean seawater, we must account for the fact that the solubility of FAP nanoparticles increases with decreasing particle size (*59*). This is a thermodynamic effect; with decreasing particle size, the proportion of surface becomes sufficiently large relative to the bulk crystal that its properties deviate accordingly (*60*). The effect of particle size on FAP solubility can therefore be determined with knowledge of its surface free energy (γ), which becomes an increasingly significant contribution to the energetics of the solid at small particle sizes. Van Cappellen’s (*61*) quantification of FAP surface free energy in seawater (289 mJ m^−2^) allows us to estimate that the solubility of 10- to 20-nm FAP particles would increase such that the corresponding seawater P concentrations would be approximately one order of magnitude higher than those associated with bulk FAP (Fig. 7), on the order of ~40 μmol/kg at pH 6.5.

We also have to consider whether FAP particles settle from the water column before particle dissolution is complete. By using laboratory FAP dissolution rates in seawater at relevant pH [i.e., 6 to 8; (*62*)], we conclude that the smallest FAP particles (on the order of 10- to 40-nm radii) would completely dissolve in 20 to 75 days (fig. S8). Accounting for errors in the dissolution rate (up to approximately one order of magnitude), this lifetime is far shorter than particle settling times expected for nanoparticulate material, which, through Stokes Law, equates to 0.18 m/year for a 100-nm FAP particle. By analogy to other nanoparticulate phases identified in modern hydrothermal plumes (*63*), these particles would be expected to reside in the oceanic water column for several years and, more likely, decades. Thus, we conclude that the preservation of FAP particles implies that seawater was at or above saturation with respect to FAP to arrest the thermodynamic drive for dissolution.

A final consideration affecting the estimates of total seawater P concentrations relates to the presence of dissolved Fe(II), which is demonstrated by the occurrence of greenalite and siderite in the jaspilites. Brady *et al.* (*64*), on the basis of new and existing solubility data for the Fe-phosphate system in seawater, showed that dissolved Fe(II) leads to strong aqueous complexing with dissolved phosphate species, in turn, increasing the solubility of all phosphate minerals. Accounting for this effect leads to even higher estimates for seawater P concentrations, which, for 10-nm FAP particles, corresponds to 10 to 60 μmol/kg (at pH 7) to 40 to 150 μmol/kg (at pH 6.5) (Fig. 7).

## DISCUSSION

### Fe plume precipitates

High–spatial resolution TEM observations show that hematite particles are subordinate to greenalite, siderite, and FAP in the 3.5-Ga jaspilites (Figs. 3 and 4). These observations suggest at least three possible scenarios: (i) the Fe(III)-oxides/hydroxides (now hematite) are primary and the Fe(II)-rich phases are diagenetic products of dissimilatory iron reduction (DIR) in the presence of bacterial organic matter; (ii) both the Fe(III)-oxides/hydroxides and Fe(II)-rich phases are primary co-depositional phases; or (iii) the Fe(II)-rich phases are primary and the hematite formed after deposition during oxidation of Fe(II)-bearing phases.

If the primary precipitate comprised solely Fe(III)-oxide/hydroxide, then it is unclear how reduction in the water column and sediment pore-waters could produce such a heterogeneous mineral assemblage, with Fe(II)-rich phases and hematite juxtaposed within <2 μm (Fig. 3, F and G). In addition, there is no textural evidence for the presence of diagenetic reduction fronts in the jaspilites. Instead, the minute particle size (typically <1 μm), random orientation, and uniform distribution of greenalite in the Dresser jaspilites resemble those observed in younger analogues (3.46-Ga to 2.45-Ga cherts and BIFs), which have the microfabric of an uncompacted mud (*65*) where laminated greenalite-rich muds are cut by shrinkage cracks and bedding-specific fractures (*9*, *66*).

In scenario 2, the co-deposition and preservation of Fe(III)-oxides/hydroxides and greenalite is difficult to reconcile with their stability under different redox conditions. Greenalite forms and is stable in anoxic solutions, whereas Fe(III)-oxides/hydroxides such as ferrihydrite convert to goethite or hematite under oxic conditions. The deposition of Fe(II)-silicates as well as the growth of early diagenetic siderite indicates that the water column and sediment pore waters were anoxic and enriched in dissolved Fe(II)(aq).

**Fig. 8.**
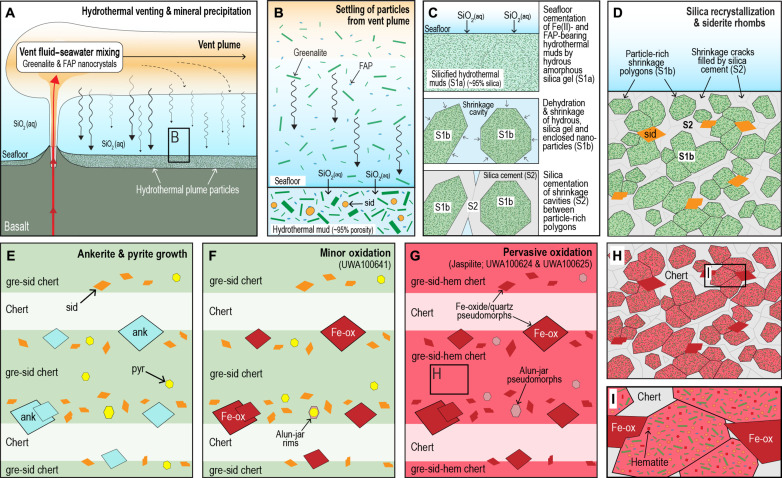
Model for the inferred origin of jaspilites from the Dresser Formation. (**A**) Hydrothermal venting of Fe^2+^ and mixing with SiO_2_-rich seawater. (**B**) Gravitational settling of greenalite and FAP from vent plumes, forming uncompacted muds with ~90 to 95% porosity. (**C**) Rapid seafloor silicification of Fe(II)-rich muds by amorphous silica cement (silica phase 1; S1a) followed by the development of shrinkage cavities as the silica gel undergoes diagenetic recrystallization (S1b). The voids between the shrunken silicified mud polygons are subsequently filled by particle-free silica cement (S2). (**D**) The development of laminae of Fe(II)-rich muds with shrinkage structures (i.e., polygons) and diagenetic siderite rhombs (sid). (**E**) Growth of ankerite-dolomite rhombs and pyrite euhedra. (**F**) Fluid-mediated oxidation of larger Fe(II)-bearing carbonate rhombs. (**G** to **I**). Pervasive oxidation of ferruginous chert and development of spectacular red pigment.

The third possibility is that the hematite was not derived from Fe(III)-oxide/hydroxide precipitates (Fig. 8). This scenario is supported by the observation that the abundance of hematite dust particles correlates with the level of background oxidation. In chert sample UWA100641, where siderite rhombs and pyrite euhedra are preserved in the dusty chert, hematite is rare, representing ≤1% of the iron-rich dust particles (Fig. 5). In contrast, hematite in jaspilite samples (UWA100624 and UWA100625), where carbonate rhombs have been completely oxidized and replaced, comprises about 20 to 30% of the iron-rich particles (Figs. 3 and 4). These observations are consistent with the formation of hematite dust particles via partial postdepositional oxidation of precursor Fe(II)-rich dust particles. In scenario 3, the preservation of greenalite and siderite nanoparticles in the most oxidized jaspilites may be explained by their isolation from oxidizing fluids within quartz crystals (Fig. 8).

There is widespread microtextural, paleomagnetic, and radiometric evidence for postdepositional hematite growth in younger ferruginous cherts and BIFs (*67*–*69*). Secondary iron-oxide replacement of Fe(II)-rich minerals is a precondition for the formation of BIF-hosted hematite ore deposits such as those in the Hamersley Province (e.g., Mt. Tom Price and Mt. Whaleback mines) (*70*, *71*) and northern Pilbara Craton (Y2/3 mine and Yarrie) (*72*). The paragenetic textures preserved in the Dresser jaspilites (Fig. 2) closely resemble textures in ferruginous chert from major Neoarchean BIFs, where dusty hematite growth in greenalite- and siderite-bearing chert has been linked to transgressive oxidation fronts (*11*, *68*, *69*). In these secondary jaspilitic bands, where hematite coexists with dusty greenalite, coarse-grained siderite, and ankerite rhombs are replaced by fine-grained hematite and magnetite, indicating postdepositional oxidation.

In the ~3.46-Ga Marble Bar Chert Member of the Warrawoona Group, which overlies the nearby Dresser jaspilites, dusty hematite has partially replaced fine-grained greenalite and siderite particles in jaspilite bands (*34*). Secondary hematite growth is also pervasive in the overlying Apex Basalt, particularly along lithological contacts, fractures, and cleavage planes (*34*). The timing of hematite alteration in the basalt is constrained to between ≥2.76 Ga, based on Re-Os dating of pyrite in veinlets that are interpreted to crosscut hematite (*73*), and ≤ 0.2 Ga, based on U-Th-Pb isotope data (*74*).

The timing of hematite growth in the Dresser Formation is unclear, although recent radiogenic Nd isotope analyses of the jaspilites indicate significant postdepositional alteration (*27*). A Sm-Nd isochron yields an age of 2260 Ga ± 180 Ma, which overlaps with known tectonothermal events in the Pilbara Craton, including the ~2.32-Ga Sylvania orogeny (*75*) and the 2.22-Ga to 2.15-Ga Ophthalmia orogeny (*76*). The ~2.26-Ga isochron age also corresponds with U-Pb xenotime ages for the onset of hematite mineralization in the 2.48-Ga to 2.45-Ga Brockman Iron Formation in the southern Pilbara Craton (2.22 Ga to 2.15 Ga; Mt. Tom Price mine) (*77*) and in the ~3.1-Ga Nimingarra Iron Formation in the northern Pilbara Craton (2.25 Ga to 2.19 Ga; Yarrie mine, Y2/3 pit, and Shay Gap 7 pit) (*72*). The proximity of high-grade hematite mineralization in BIFs and jaspilites immediately west of the North Pole Dome (e.g., Abydos and Miralga Creek), along a major NE-SW structural zone, offers a plausible explanation for the origin of some of the hematite in the Dresser jaspilites.

In conclusion, the nanoscale observations and comparative studies with younger BIFs and ferruginous cherts favor scenario 3 (Fig. 8), in which iron released from seafloor vents was deposited as greenalite. The greater abundance of fine-grained hematite in chert that has undergone secondary oxidation suggests that some or perhaps all the hematite formed after burial. This scenario is consistent with geochemical modeling of hydrothermal alteration of basaltic crust by anoxic, sulfate-free solutions simulating Archean seawater, which shows that the main Fe-bearing mineral precipitates during hydrothermal venting are greenalite, siderite, and pyrite (*13*). The quantity of Fe(II)(aq) partitioned into Fe(II)-rich phases from aqueous fluids is up to 96 to 99%, consistent with nanoscale observations that the primary plume particles comprise almost entirely greenalite and possibly siderite (scenario 3).

### Implications for BIF deposition

The hydrothermal precipitation of greenalite and FAP near seafloor vents may have implications for BIFs depositional models. Previous studies have largely focused on the scenario that greenalite and FAP preserved in 2.6-Ga to 2.4-Ga BIFs precipitated in the water column near the depositional environment from distal hydrothermal plumes (*10*, *78*). However, the simultaneous high-temperature growth of greenalite and FAP during hydrothermal fluid–seawater mixing, combined with their minute size and low specific gravity, suggests that both phases may have formed during venting and traveled vast distances (thousands of kilometers) as nanoparticles before deposition. As a consequence, the precursor sediments of BIFs may comprise greenalite and FAP that formed in two distinct environments (vent proximal and distal shelves) under different geochemical conditions. If correct, then the depositional site would have been much larger, encompassing areas between the seafloor vents and the continental shelf.

Although greenalite and FAP particles are not major phases in BIFs today, being mostly restricted to laminated chert from minimally deformed and metamorphosed sequences, they are texturally the earliest phases preserved in BIFs (*11*, *79*, *80*). On the basis of their physical and chemical characteristics, they are interpreted to be relicts of once vast distal plume deposits that were preserved from fluid-mediated recrystallization by very early diagenetic silica cement. After deposition, greenalite that was not encased in chert was apparently replaced by authigenic minerals in response to diagenesis (siderite, stilpnomelane, and silica), metamorphism and metasomatism (magnetite, minnesotaite, riebeckite, stilpnomelane, and ankerite-dolomite), and hydrothermal oxidation and weathering following exhumation (goethite, hematite, and magnetite).

The preservation of greenalite and FAP particles in laminated chert ranging in age from 3.5 Ga to 2.4 Ga and from diverse environments, from volcanic-associated vent-proximal environments to distal settings, and from deepwater slopes to shallow platforms indicates that vent-plume deposition of greenalite and FAP was likely a pervasive process operating across the marine realm. While deposition around hydrothermal vents was likely semicontinuous, the episodic encroachment of distal hydrothermal plumes onto continental margins and shelves may have coincided with peaks in global mafic/ultramafic magmatism and volcanism. We argue that greenalite and FAP deposition from hydrothermal plumes was a major source of the precursor sediments of BIFs and, more broadly, was an important sink for hydrothermal Fe and P released into the early oceans from hydrothermally altered basalt-dominated crust.

### Role of biology in iron deposition

Evidence from the rock record for biological Fe oxidation in the Paleoarchean relies on the assumption that fine-grained hematite in jaspilites and BIFs is derived from primary Fe(III)-oxide/hydroxide precipitates. The rationale is that, in an anoxic atmosphere and ocean, photochemical oxidation of Fe^2+^(aq) (*81*) was negligible (*15*, *82*), requiring biological oxidation either by anoxygenic phototrophic bacteria (*83*), microaerophilic bacteria, or Cyanobacteria (*14*, *84*).

However, if our nanoscale microtextural observations and geochemical modeling are correct, then the assumption that hematite is the primary Fe-phase of jaspilites is flawed. A secondary origin for hematite (Fig. 8) would negate the requirement for biological Fe oxidation in the water column and DIR in the hydrothermal sediments. While more work is needed to establish the timing of hematite growth in Paleoarchean jaspilites, its presence in red banded chert from the Dresser Formation should not be used as unequivocal evidence for an active biological iron cycle.

### Implications for the Archean P cycle

**Fig. 9.**
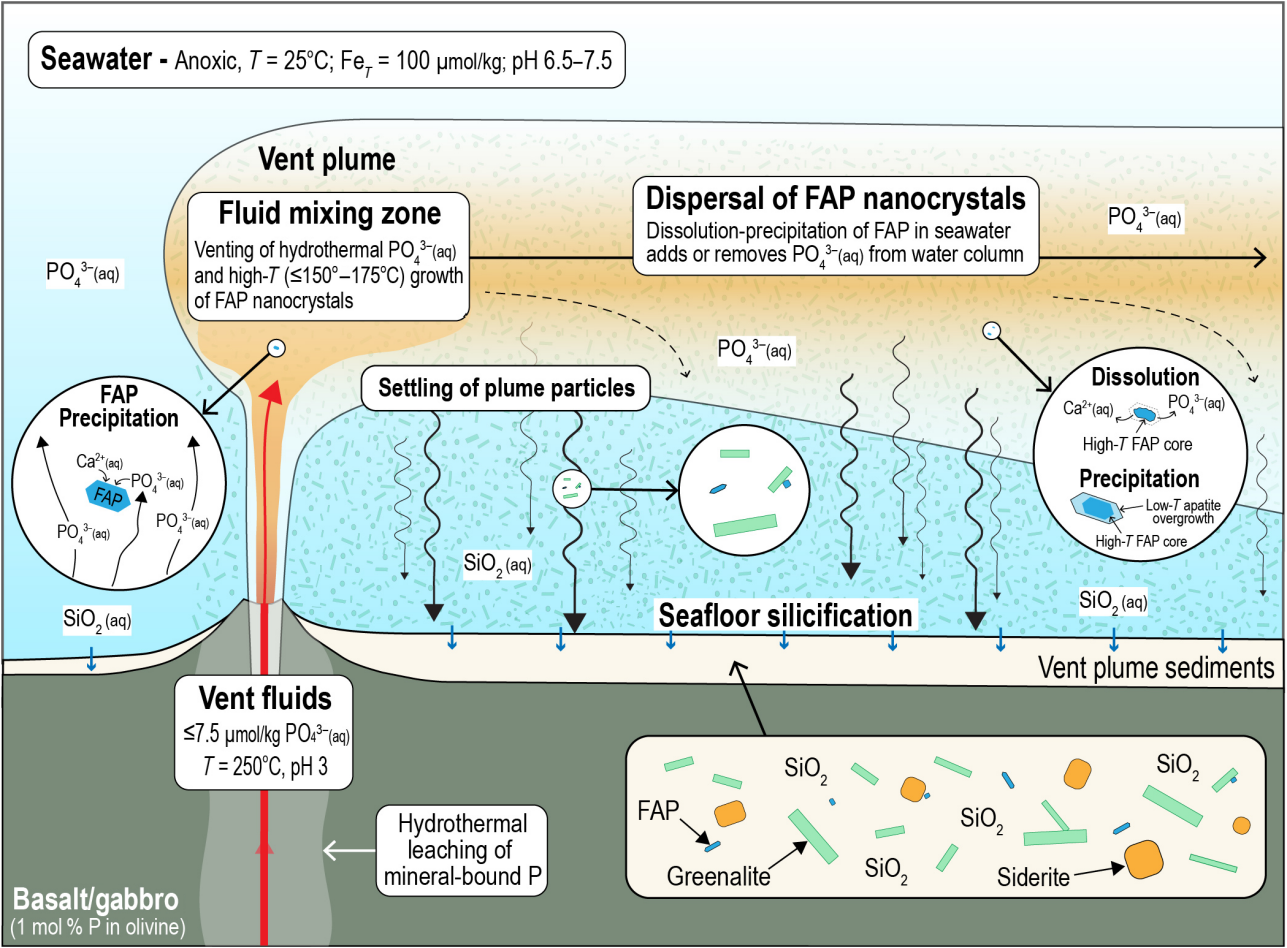
Depiction of Paleoarchean P cycle in basalt-hosted vent system. The hydrothermal alteration of basaltic lavas and gabbroic intrusions by sulfate-free anoxic seawater liberates mineral-bound P into ascending fluids in upflow zones. At temperatures between 150° and 175°C, about half of the dissolved P precipitates as FAP during mixing between hydrothermal fluids and seawater. As hydrothermal FAP nanoparticles are dispersed across the ocean, they may undergo either dissolution, releasing dissolved P, or low-temperature overgrowth, removing dissolved P from the water column. The non-dissolution of FAP nanoparticles as small as 10 nm in solutions enriched in Fe^2+^(aq) implies dissolved P concentrations of between 10 and 60 μmol/kg at pH 7 or between 40 and 150 μmol/kg at pH 6.5. Geochemical parameters represent conditions investigated and/or derived from reaction path models of hydrothermal vent fluid–seawater mixing.

Our nanoscale observations show that P in exhalative cherts from the 3.5-Ga Dresser Formation is hosted in FAP nanoparticles that are distributed among larger greenalite and siderite particles and enclosed in chert cement. Our reaction path modeling suggests that at least some of the P in FAP precipitates was derived from hydrothermal leaching of mineral-bound P in submarine tholeiitic basalts (Fig. 9).

Because FAP is normally very insoluble, its precipitation would theoretically scrub most of the P liberated during basalt leaching from the fluid. However, because of the effects of dissolved Fe^2+^ and small particle size, the FAP that is formed is much more soluble than simple equilibrium conditions suggest. This means that not all of the leached P was precipitated and that some escaped into the ocean. Although the amount varies depending on a number of factors, our calculations indicate that it is possible that up to half of the leached P is vented into the ocean. This implies that under Archean conditions, basalt-hosted seafloor vents were more likely a source of dissolved P rather than a sink.

The introduction of FAP nanoparticles into an ocean initially undersaturated with respect to FAP would cause it to dissolve and supply P to seawater until saturation is reached. Above saturation levels, there will be a thermodynamic driving force for low-temperature FAP growth upon FAP seeds, leading to the removal of dissolved P from seawater (Fig. 9). The similar abundance and size distribution of FAP particles in younger BIFs deposited in vent-distal settings (*79*, *80*) suggests that seawater FAP growth, rather than dissolution, dominated in the Archean and early Paleoproterozoic oceans during BIF deposition. The carbonate-associated phosphate proxy also implies that dissolved seawater P was elevated during the deposition and cementation of early Precambrian carbonates (*85*).

Constraining the concentration of seawater P in the Archean ocean has traditionally used the P content of BIFs and jaspilites (*15*, *86*–*88*). Their use as a P proxy is based on the observation that modern vent-derived Fe^2+^ is rapidly oxidized to Fe(III)-oxide/hydroxide particles upon mixing with oxygenated seawater, scavenging dissolved P from the water column (*3*, *89*). The quantitative stripping of P via adsorption onto oxide particle surfaces is observed in the modern water column (*2*, *3*) and is also preserved in Miocene hydrothermal sediments from the East Pacific Rise (*4*). On the assumption that hematite and magnetite in BIFs and jaspilites are primary phases, the removal of dissolved seawater P by iron oxides during deposition has been used to estimate the P content of seawater. This approach, however, is not applicable to the jaspilites of the Dresser Formation if the hydrothermal Fe was deposited principally as Fe(II)-rich phases, as suggested here.

Alternatively, P concentrations can be constrained if, as our observations suggest, FAP nanoparticles precipitated and were not dissolved in mildly acidic to neutral seawater enriched in Fe^2+^. For instance, the preservation of 10-nm particles indicates P concentrations of between 10 and 60 μmol/kg at pH 7 or between 40 and 150 μmol/kg at pH 6.5 (Fig. 7). These values are at least five times higher than modern deep sea P concentrations (~2 μmol/kg). If the shallow water depths (<100 m) (*21*, *23*) during deposition of the Dresser Formation are correct, then hydrothermal FAP particles were emitted directly into the photic zone, implying that seawater P concentrations were many orders of magnitude higher than in today’s surface ocean, where dissolved inorganic P concentrations are extremely low due to rapid biological consumption. The inferred high concentrations of dissolved P in the 3.5-Ga photic zone imply that biological P uptake was relatively low, perhaps reflecting reduced primary productivity in a marine biosphere dominated by anoxic phototrophic metabolisms (*90*). This scenario is consistent with recent suggestions that seawater P concentrations may have been higher before ~2.4 Ga (*79*, *80*, *85*, *91*), challenging the paradigm of a phosphate-limited Archean biosphere (*92*).

In summary, our results imply that hydrothermal circulation of Archean seawater through tholeiitic basalts liberated mineral-bound P (Fig. 9). Upon mixing with seawater, at least half of the dissolved P precipitated as FAP nanocrystals, some of which were deposited with greenalite from vent plumes and silicified on the seafloor. The preservation of minute FAP particles in 3.5-Ga exhalites suggests that P concentrations were >10 μmol/kg for neutral seawater or >40 μmol/kg for slightly acidic seawater. Their wider dispersal beyond vent-proximal settings would have rendered FAP nanoparticles either a sink and/or source of dissolved P depending on whether authigenic growth or particle dissolution dominated in the water column. We speculate that hydrothermal FAP nanoparticles likely played an important role in regulating P concentrations in the early oceans, especially before the development of a nutrient-limited biosphere.

### Primordial vent plumes as sites for template-directed biosynthesis

Seafloor hydrothermal vent systems are one of several environments favored for the emergence of life (*93*–*95*). As the oldest well-preserved volcano-sedimentary sequence with exhalative deposits, the Dresser Formation provides important clues about submarine hydrothermal environments 3.5 Ga ago but also offers a window into an earlier world before life that was dominated by basaltic volcanism and seafloor hydrothermal alteration and venting. For this reason, the identification of the products of hydrothermal vent fluid–seawater mixing has implications for understanding the range of mineral surfaces available for template-directed reactions, dissolved P availability in vent fluids and seawater, and the conditions and processes operating in a prime site for the emergence of life on Earth.

According to the “RNA world” hypothesis, DNA- and protein-based life was preceded by a simpler life form based primarily on RNA, which carried genetic information and acted as a catalyst for biochemical reactions (*19*, *96*, *97*). For RNA molecules to hold information and serve as catalysts, longer chains in the range of 30 to 60 monomers were required (*18*). A central problem for the RNA world hypothesis is to explain how dilute solutions of nucleic acid monomers assembled into polymers without protein enzymes or ribozymes. One possibility is that mineral templates promoted polymerization (*96*, *98*–*100*). This idea has support from experiments showing that the presence of very fine-grained montmorillonite and hydroxylapatite promotes the assembly of activated monomers of nucleotides and amino acids, respectively (*18*).

If mineral surfaces were involved in prebiotic synthesis in hydrothermal environments, then greenalite and FAP are likely candidates because both minerals were probably forming continuously in primordial oceans around basalt-hosted seafloor vents. In comparison to other minerals, greenalite may have been favorably preconfigured as an information-rich template, with a regular yet aperiodic crystal structure characterized by parallel grooves ~2.2-nm wide and between 30- and 500-nm long (*101*) along its edges. The width of the grooves (~2.2 nm) is similar to the diameter of helical strands of DNA (~2.0 nm), whereas their minimum length along crystal edges (~30 nm) (Fig. 5M) would have been sufficient to polymerize long chains of >30 monomers. These linear, parallel grooves, which were theorized to be an important feature of the idealized mineral substrate (*99*, *100*, *102*), are potentially the right size and shape to orient and guide monomers into position, lowering the activation energy barrier required to form inter-monomer bonds. Given its incommensurate modulated crystal structure, the presence of Ångstrom-scale variations in surface charge density in grooves may have helped to specify nucleotide monomers. It is conceivable that the addition of nucleoside triphosphates onto growing polynucleotides aligned in the greenalite grooves may also have released energy, thereby combining primitive metabolism with the assembly of RNA-like polymers.

The formation of FAP (or other apatite group minerals) offers a second distinct mineral template with phosphate-rich surfaces (*103*). In contrast to montmorillonite, laboratory experiments suggest that fine-grained hydroxylapatite can catalyze the formation of longer chains (up to 55 mer) of amino acids (*18*). The formation of Ca-phosphate particles also indicates that the vent plume-seawater mixtures were enriched in dissolved phosphate (see Fig. 7), which is an important precondition for biosynthesis. In magma-hosted hydrothermal systems, some of the P species may have included polyphosphates, whose origin on prebiotic Earth was high-temperature dehydration and condensation of monophosphates during volcanic activity (*104*). Polyphosphates not only are a source of P but also are high-energy molecules that likely preceded RNA, DNA, and proteins in prebiotic evolution (*105*).

Another question plaguing the RNA world hypothesis is: Where did all the RNA come from? Prebiotic reactions preceding the formation of RNA were likely messy and generated a diverse range of heterogeneous molecules. Crick (*96*) speculated that, if a natural mineral catalyst for random nucleotide polymerization did exist, “RNA may have been made at very many places on the earth’s surface over a very considerable amount of time, so that altogether an enormous number of different sequences may have been synthesized.” For example, for RNA molecules comprising 40 nucleotides, which is within the range of minimum sizes for enzymatic activity, there are 4^40^ (1.2 × 10^24^) possible sequences (*19*).

The formation of greenalite- and FAP-rich plumes above basalt-hosted hydrothermal vents in primordial oceans represents a template-rich environment with an enormous surface area–to–volume ratio. On the basis of the distribution of greenalite particles in FIB foils (average of 25 nanoparticles in 8 μm by 8.5 μm by 1 μm volume), a cubic centimeter of dusty chert is estimated to have contained ~367 billion greenalite nanoparticles. Assuming that the iron-bearing banded cherts of the Dresser Formation were ~3-m thick and limited to an area of 20 km^2^ (e.g., eastern belt of the North Pole Dome), we estimate that at least 22 trillion trillion (22 × 10^24^) greenalite nanoparticles were deposited. Given the abundance of grooves along the edge of each crystal (up to ~450 along a 1-μm-long edge), the number of sequences that could have been assembled is likely to be several orders of magnitude higher. In addition, our calculations show that an even larger number of FAP nanoparticles were produced, yielding a total of >50 trillion trillion nanoparticles. Given the vast number of free-floating catalytic templates, an enormous number of random sequences of RNA-like polymers and polypeptides could have been synthesized, some of which may have had traits suitable for replication and catalysis, thereby helping to overcome the improbability of life emerging from simple building blocks.

If polymers of nucleic and amino acids assembled on mineral surfaces, then their compartmentalization was a prerequisite for the development of self-replicating protocells. Simple vesicles comprising amphiphilic molecules such as fatty acids, perhaps derived from carbonaceous chondrites (*106*) or produced by abiotic organic synthesis in hydrothermal vent systems (*33*, *107*), could have self-assembled into stable vesicles. Experiments have shown that fine-grained montmorillonite particles can accelerate the spontaneous conversion of fatty acid micelles into vesicles and, in some cases, encapsulate clay particles with adsorbed RNA (*108*). Because some of the greenalite crystals are only a few hundred nanometers long, they may have become encapsulated within fatty acid vesicles (1 to 2 μm in diameter), providing a mechanism for trapping catalytic templates with RNA polymers within semipermeable membranes [cf. (*108*, *109*)]. The encapsulation of FAP nanoparticles (50- to 300-nm long) may also have provided a second catalytic template for polymerization as well as a source of bioavailable P within primitive cell membranes.

## MATERIALS AND METHODS

### Optical microscopy

Polished thin sections (~30-μm thick) were prepared and examined by optical and scanning electron microscope. Routine optical microscopy, using transmitted and reflected plane-polarized light, was carried out on polished thin sections to collect mineralogical and textural information about the jaspilite and banded chert samples from the North Pole area.

### Focused ion beam

Lamellae for TEM analyses were cut from polished thin sections of jaspilite and gray-green banded chert from the Dresser Formation (North Pole Dome, Western Australia). FIB techniques were used to prepare ~100-nm-thick TEM lamellae using an FEI Helios NanoLab G3 CX DualBeam instrument located at Centre for Microscopy, Characterisation and Analysis (CMCA), University of Western Australia (UWA). The areas selected for TEM analysis were first coated with a strip of Pt, 2-μm thick to protect the surface, and then trenches 7-μm deep were milled on either side of the strip using a gallium ion beam with 30-kV voltage and 9.3-nA current. The lamellae were then cut away from the samples and welded to Cu TEM grids. The lamellae were thinned with the gallium ion beam at 30 kV and 0.79 and 0.23 nA, before cleaning at 5 kV and 41 pA, and polishing at 2 kV and 23 pA.

### Transmission electron microscopy

TEM data were obtained at 200 kV using an FEI Titan G2 80–200 TEM/scanning TEM (STEM) with ChemiSTEM technology located at CMCA, UWA. Bright-field TEM and STEM, high-resolution TEM, and high-angle annular dark-field STEM images were collected and processed using TIA (TEM Imaging and Analysis) software from FEI. Qualitative EDS spectra and maps were collected with an FEI Super-X EDS detector and processed using Esprit software from Bruker Corporation.

### Thermodynamic data and geochemical modeling

Geochemical reaction path models were implemented with the React module of The Geochemists Workbench v2020 (*110*). Thermodynamic data for these calculations were generated with the Python module pyGCC (*111*), which is a consolidated set of functions for calculating the thermodynamic properties of gas, aqueous, and mineral species (including solid solutions and variable-formula clay minerals) and of reactions between these species, over a broad range of temperature and pressure conditions. Modifications to thermodynamic data and details of calculations from which thermodynamic data were generated are described by Tosca and Tutolo (*13*).

The solubility of FAP as a function of particle size was estimated by modifying the expression for the solubility product to account for the Gibbs free energy contribution from the bulk solid and from the surface free energy, yielding the following expression

$$\text{log}\;K_{\text{sp}\left(S\right)}=\text{log}\;K_{\text{sp}\left(S=0\right)}+\frac{\frac{2}{3}\gamma }{2.3\mathrm{RT}}S$$

where *K*_sp(*S*)_ is the solubility product at a given specific surface area, *K*_sp(*S*=0)_ is the solubility of bulk FAP, γ is the surface free energy, *R* is the gas constant, *T* is the temperature, and *S* is the molar surface area.
