# Supplementary material for: Nanoparticulate apatite and greenalite in oldest, well-preserved hydrothermal vent precipitates
Source: Sci Adv. 2024 Jan 26;10(4):eadj4789. doi: 10.1126/sciadv.adj4789 (PMC10816723; doi:10.1126/sciadv.adj4789)
Supplement: Supplementary file 1 — Figs. S1 to S8 [file sciadv.adj4789_sm.pdf]

Science Advances
AAAS

Supplementary Materials for

Nanoparticulate apatite and greenalite in oldest, well-preserved hydrothermal vent precipitates

Birger Rasmussen *et al.*

Corresponding author: Birger Rasmussen, birger.rasmussen@uwa.edu.au

Sci. Adv. **10**, eadj4789 (2024)
DOI: 10.1126/sciadv.adj4789

This PDF file includes:

Figs. S1 to S8

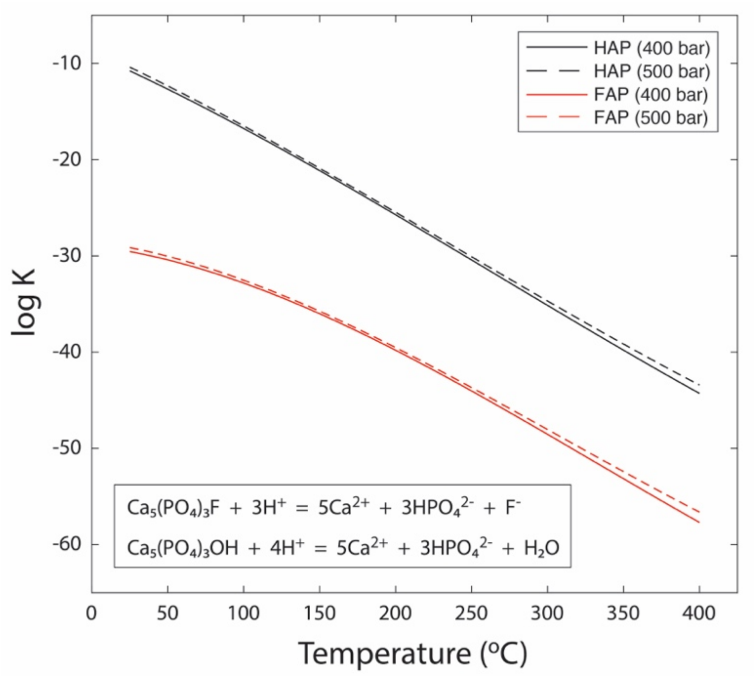

Figure S1. Equilibrium constants for the hydroxyapatite (HAP) and fluorapatite (FAP) precipitation reactions as a function of temperature at 400 and 500 bar (calculated using methods described in the main text and data by Zhu and Sverjensky (*54*). In general, both minerals become significantly less soluble with increases in temperature; in comparison, pressure effects are relatively minor.

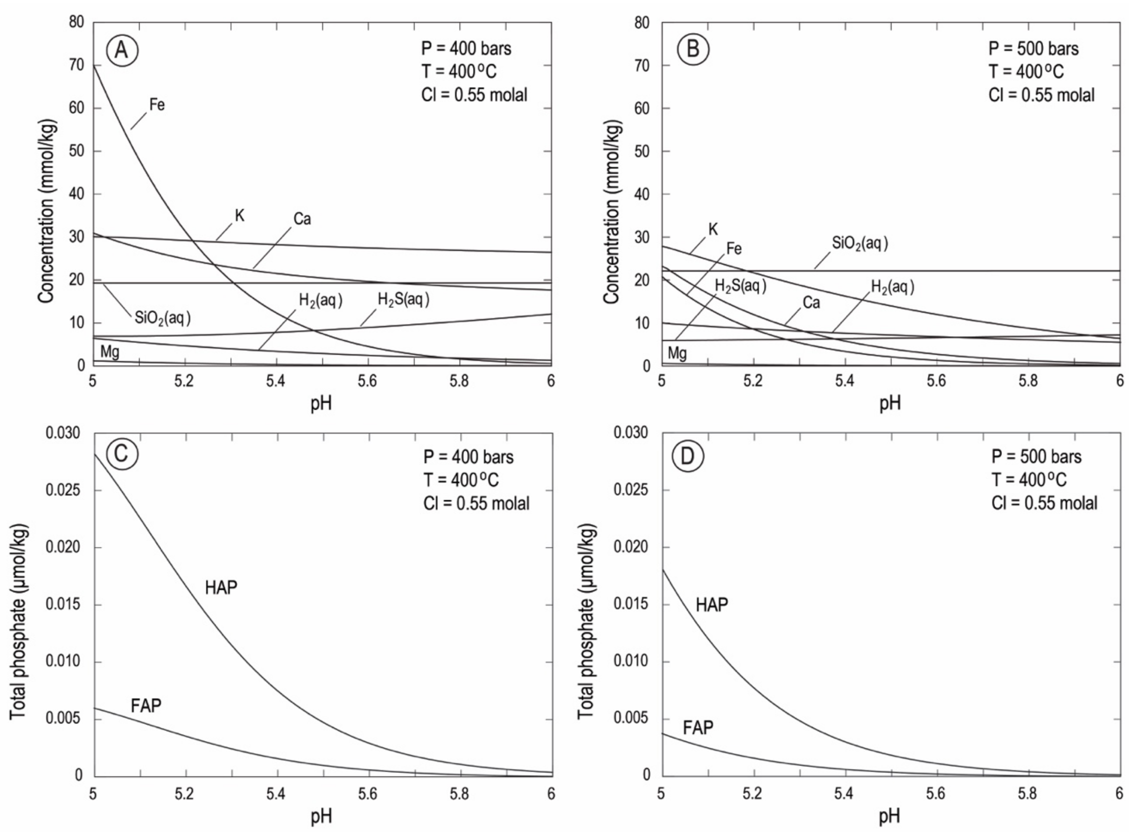

Figure S2. Equilibrium fluid chemistry predicted for the Na_2O-K_2O-CaO-MgO-FeO-Fe_2O_3-Al_2O_3-SiO_2-P_2O_5-H_2O-HCl-H_2S-HF system in the presence of plagioclase solid solution, epidote solid solution, clinochlore, K-feldspar, quartz, fayalite, pyrrhotite, magnetite, either fluorapatite (FAP) or hydroxyapatite (HAP) and aqueous fluid at 400°C and (A) 400 bar and (B) 500 bar. Total phosphate concentrations at 400 bar (C) and 500 bar (D) are controlled by either FAP or HAP equilibria.

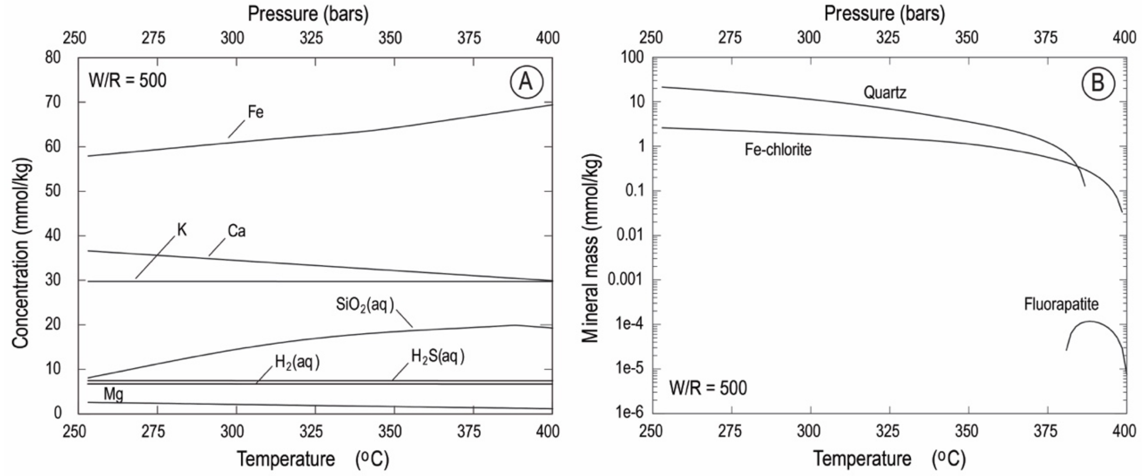

Figure S3. Calculated fluid chemistry (A) and mineral mass precipitated (B) during the cooling and decompression of reaction zone fluids derived from the equilibration between SO_4-free seawater and basalt/gabbro at 400°C and 400 bar and in situ pH = 5 (corresponding to fluid composition under solubility control with FAP in Fig. S2). To simulate reaction with wall rock during fluid upflow, calculations included the reaction of fresh crystalline basalt/gabbro containing olivine with a total P concentration of 1 mol % at a water/rock mass ratio of 500.

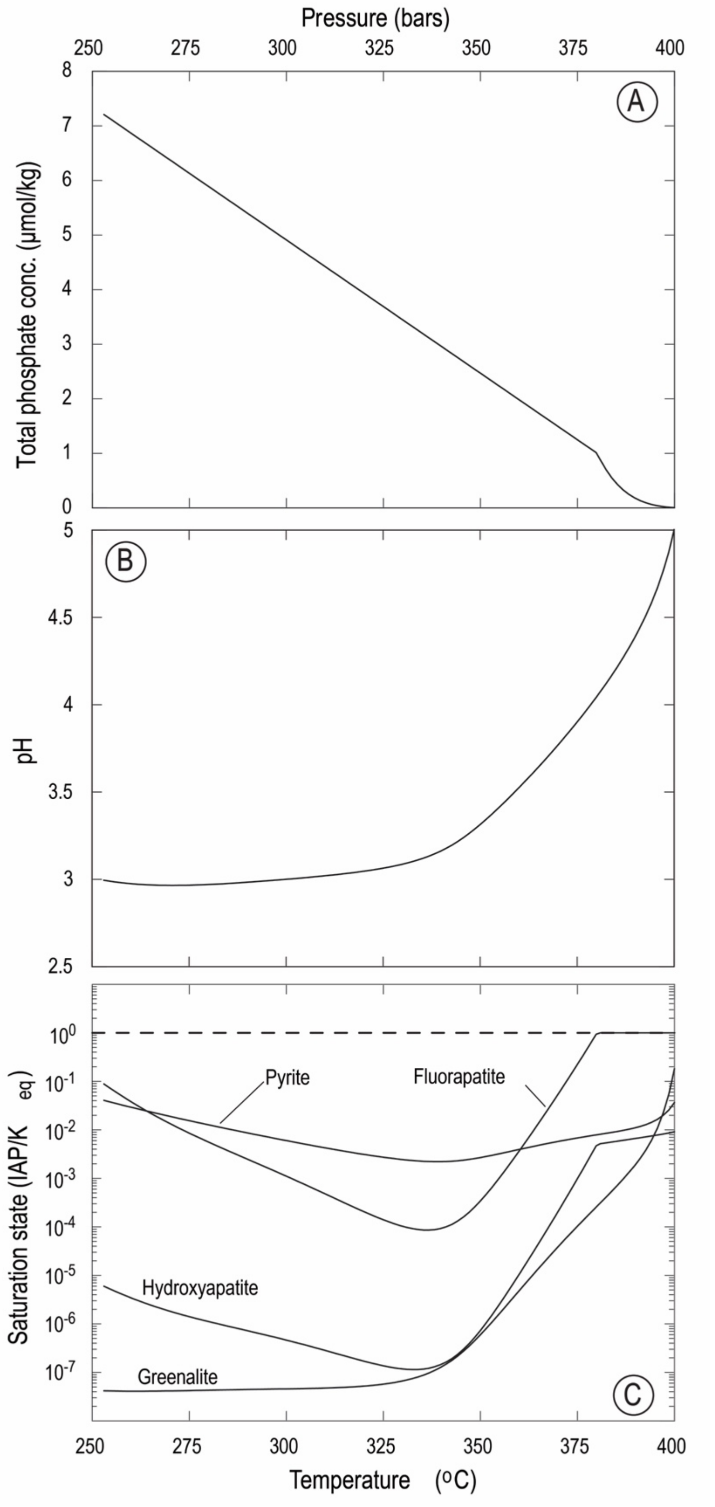

Figure S4. Calculated concentration of total dissolve phosphate (A), *in situ* pH (B), and the saturation state (C) of relevant minerals during the cooling and decompression of reaction zone fluids derived from the equilibration between SO_4-free seawater and basalt/gabbro containing olivine with a total P concentration of 1 mol % at 400°C and 400 bar and *in situ* pH = 5 (corresponding to fluid composition in Fig. S2). To simulate reaction with wall rock during fluid upflow, calculations included the reaction of fresh crystalline basalt/gabbro containing olivine with a total P concentration of 1 mol % at a fluid/rock mass ratio of 500.

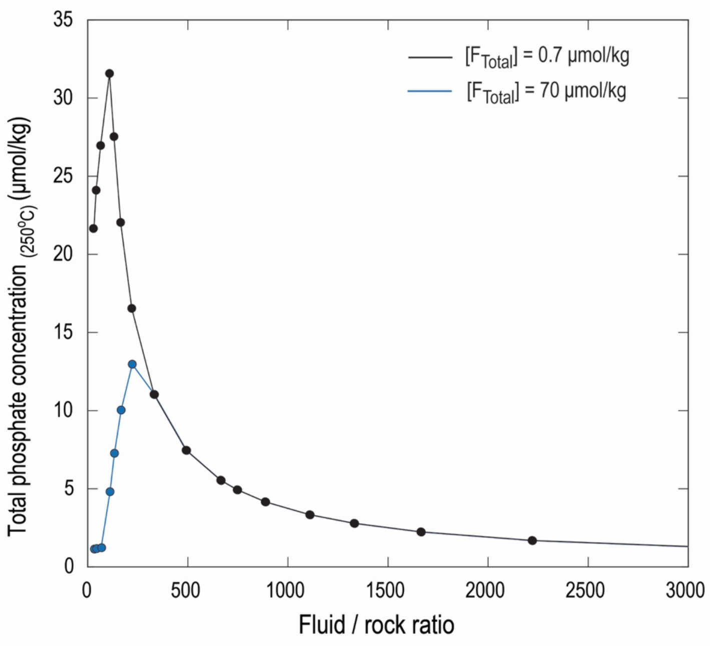

Figure S5. Effect of fluid/rock mass ratio on calculated total dissolved phosphate concentration at 250°C after cooling and decompression of reaction zone fluids derived from the equilibration between SO_4-free seawater and basalt/gabbro at 400°C and 400 bar and *in-situ* pH = 5 (corresponding to fluid composition in Fig. S2). At low fluid/rock ratios, silicate hydrolysis buffers *in-situ* pH to higher values, which promotes late-stage apatite precipitation, depending on total F concentration. This results in a decrease of total dissolved phosphate concentration from a maximum which corresponds to the highest concentration achieved before late-stage apatite precipitation occurs at lower fluid/rock ratio. At fluid/rock ratios above this, total phosphate concentration is dictated by the P content of the basalt, the mass of P-containing basalt hydrolysed, and fluid/rock ratio.

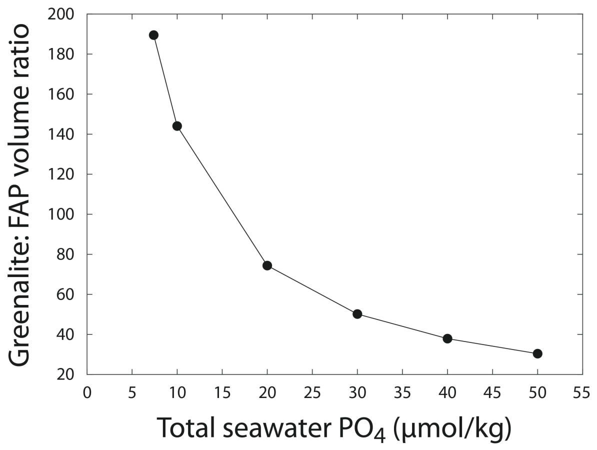

Figure S6. Calculated volume ratio between greenalite and FAP in mineral products generated in response to mixing between hydrothermal fluids and seawater. The calculations correspond to those shown in figures S3A and S4A. The calculations show that increases in the total concentration of seawater phosphate result in increases in the proportion of FAP generated relative to greenalite upon mixing. As discussed in the text, apatite reprecipitation within hydrothermal fluids ascending through the oceanic crust limit effective total P concentrations to approximately 7.5 µmol/kg under these conditions.

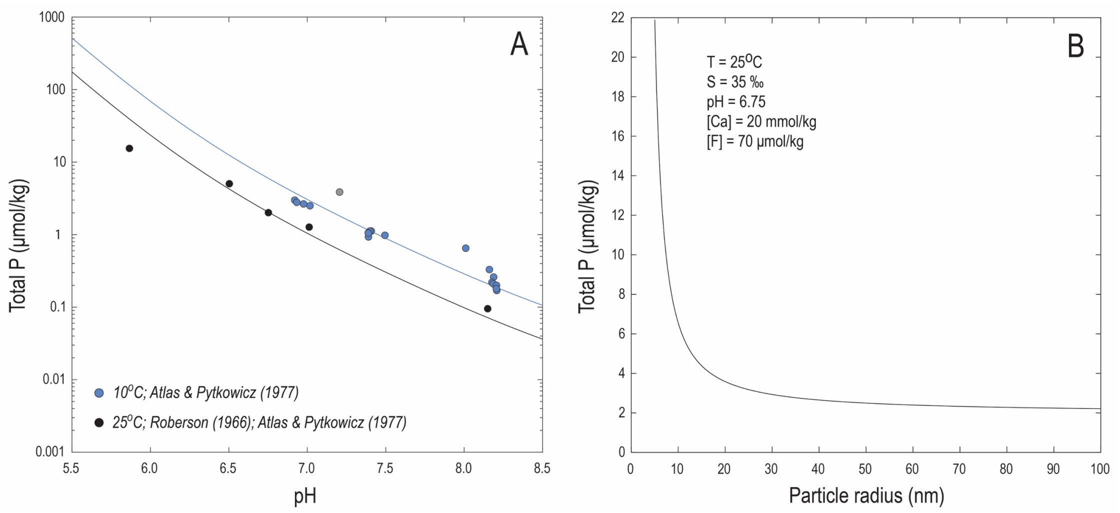

Figure S7. (A) Laboratory measurements of crystalline FAP solubility in modern SW for 25°C (Roberson, [*55*]; Atlas and Pytkowicz [*56*]) and 10°C (Atlas and Pytkowicz [*56*]). (B) Plot showing the relationship between apatite particle size and calculated total dissolved phosphate concentration at 25°C and pH 6.75.

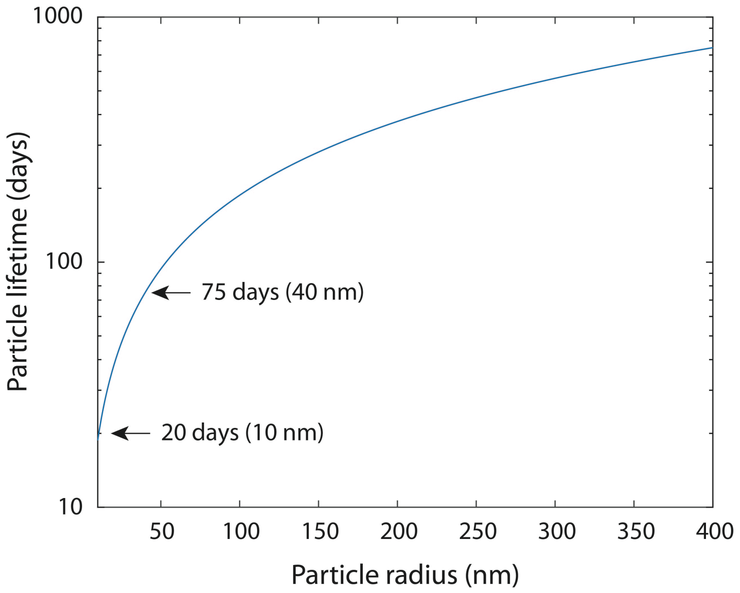

Figure S8. Calculated lifetime of FAP particles in seawater as a function of particle radius at 25°C. Lifetime calculations were derived using surface-area normalised dissolution rates for FAP in modern seawater at pH 6-8 (Guidry and Mackenzie [*62*]) and molar volume of FAP. These constraints imply that the smallest FAP nanoparticles would dissolve in 20-75 days, which is significantly less than their estimated residence time in seawater based on Stokes law particle settling velocities.
